# DNA Barcoding to Improve the Taxonomy of the Afrotropical Hoverflies (Insecta: Diptera: Syrphidae)

**DOI:** 10.1371/journal.pone.0140264

**Published:** 2015-10-16

**Authors:** Kurt Jordaens, Georg Goergen, Massimiliano Virgilio, Thierry Backeljau, Audrey Vokaer, Marc De Meyer

**Affiliations:** 1 Department of Biology–Invertebrate Section and Joint Experimental Molecular Unit (JEMU), Royal Museum for Central Africa, Leuvensesteenweg 13, B-3080 Tervuren, Belgium; 2 Department of Biology, University of Antwerp, Groenenborgerlaan 171, B-2020 Antwerp, Belgium; 3 International Institute of Tropical Agriculture, 08 BP 0932 Tri Postal, Cotonou, Republic of Benin; 4 Royal Belgian Institute of Natural Sciences–OD Taxonomy and Phylogeny and Joint Experimental Molecular Unit (JEMU), Vautierstraat 29, B-1000 Brussels, Belgium; University of Milan-Bicocca, ITALY

## Abstract

The identification of Afrotropical hoverflies is very difficult because of limited recent taxonomic revisions and the lack of comprehensive identification keys. In order to assist in their identification, and to improve the taxonomy of this group, we constructed a reference dataset of 513 COI barcodes of 90 of the more common nominal species from Ghana, Togo, Benin and Nigeria (W Africa) and added ten publically available COI barcodes from nine nominal Afrotropical species to this (total: 523 COI barcodes; 98 nominal species; 26 genera). The identification accuracy of this dataset was evaluated with three methods (K2P distance-based, Neighbor-Joining (NJ) / Maximum Likelihood (ML) analysis, and using SpeciesIdentifier). Results of the three methods were highly congruent and showed a high identification success. Nine species pairs showed a low (< 0.03) mean interspecific K2P distance that resulted in several incorrect identifications. A high (> 0.03) maximum intraspecific K2P distance was observed in eight species and barcodes of these species not always formed single clusters in the NJ / ML analayses which may indicate the occurrence of cryptic species. Optimal K2P thresholds to differentiate intra- from interspecific K2P divergence were highly different among the three subfamilies (Eristalinae: 0.037, Syrphinae: 0.06, Microdontinae: 0.007–0.02), and among the different general suggesting that optimal thresholds are better defined at the genus level. In addition to providing an alternative identification tool, our study indicates that DNA barcoding improves the taxonomy of Afrotropical hoverflies by selecting (groups of) taxa that deserve further taxonomic study, and by attributing the unknown sex to species for which only one of the sexes is known.

## Introduction

Syrphidae (hoverflies or flower flies) is one of the most diverse, and well-known to the general public, family of Diptera, with more than 6,000 species worldwide [1]. Adult hoverflies are often important for the pollination of flowering plants [2,3]. The larvae, in turn, are often notorious predators of aphids or larvae of other insects [4], and as such are useful for insect pest control e.g. [5]. Finally, some syrphid larvae are also used for weed control e.g. [6].

The Afrotropical region (i.e. Africa south of the Sahara) harbors approximately 600 species of hoverflies of three out of the four hoverfly subfamilies (viz. Microdontinae, Eristalinae and Syrphinae) [1,7,8,9]. The subfamily Pipizinae [10] is not represented on the African continent. The identification of Afrotropical hoverflies is difficult and challenging for two major reasons. First, Whittington [8] calculated that, using identification keys, it is only possible to key to species about 60% of the known fauna, and very little progress in improving identification keys has been made ever since. The larger the genus, the less reliable the identification, because there are greater chances that the keys will not work and it becomes more difficult to use the original descriptions for identification (since there are a greater number of choices) [8]. Second, many of the original descriptions are too brief or too vague for species identification, and several keys are of males only (e.g. Hull's [11] key to *Eumerus*). Very few groups have been the subject of a thorough revision, providing more detailed redescriptions. The currently available identification tools, based solely on morphological information, are therefore inadequate. The development of an accurate and effective molecular identification system would be helpful to assist morphological identification of Afrotropical hoverflies, and ecological studies on them.

DNA barcoding has become a popular and practical method for distinguishing species using a short DNA sequence from a specific locus in the genome. In most animal groups, the standard is a 658 base pair (bp) fragment of the mitochondrial cytochrome *c* oxidase subunit I (COI) gene [12,13]. Intraspecific COI barcode divergences are usually much lower than interspecific ones, a pattern often referred to as the “barcode gap”. This makes it generally straightforward to match unknown specimens to reference sequences e.g. [14,15,16], but see [17,18]. Specifically, DNA barcoding may complement, and stimulate, taxonomic research since it 1) highlights taxa that deserve further taxonomic study, especially those that exhibit high intra- or low interspecific sequence divergence, 2) links sexes in species were only one sex is known, and 3) relates developmental stages of species for which the reproductive ecology is poorly understood [15,19].

So far, only two studies dealt with DNA barcoding of Syrphidae, viz. one on West Palaearctic *Pandasyopthalmus* [20] and one on the genus *Merodon* from Lesvos Island (Greece) [21], although COI barcodes have been used in several taxonomic studies of Syrphidae e.g. [22,23,24]. Nevertheless, there are many COI barcodes/sequences in GenBank (a search with the keywords "Syrphidae and barcode" or "Syrphidae and COI" on 2 September 2015 retrieved 1,328 and 2,841 records, respectively) and in the Barcoding of Life Database System (BOLD; http://www.barcoding-life.org) [25] there are 21,621 Syrphidae specimens with barcodes (6,083 are public records with species names). Yet, there are only ten >550 bp COI barcodes from nine nominal species in GenBank or publicly available in BOLD that are from hoverflies that are identified to the species level and that occur in the Afrotropics (apart from some entries that have genus names only), and another 17 partial (i.e. 307–427 bp) COI barcodes from 12 morphospecies are available in GenBank (S2 Table; note that the *Paragus tibialis* barcodes were from European specimens but that this species also occurs in the Afrotropics). This means that species assignments for >98% of the Afrotropical hoverflies cannot be verified with the BOLD Identification System (BOLD-IDS) [25] or GenBank’s BLAST [26].

**Fig 1:**
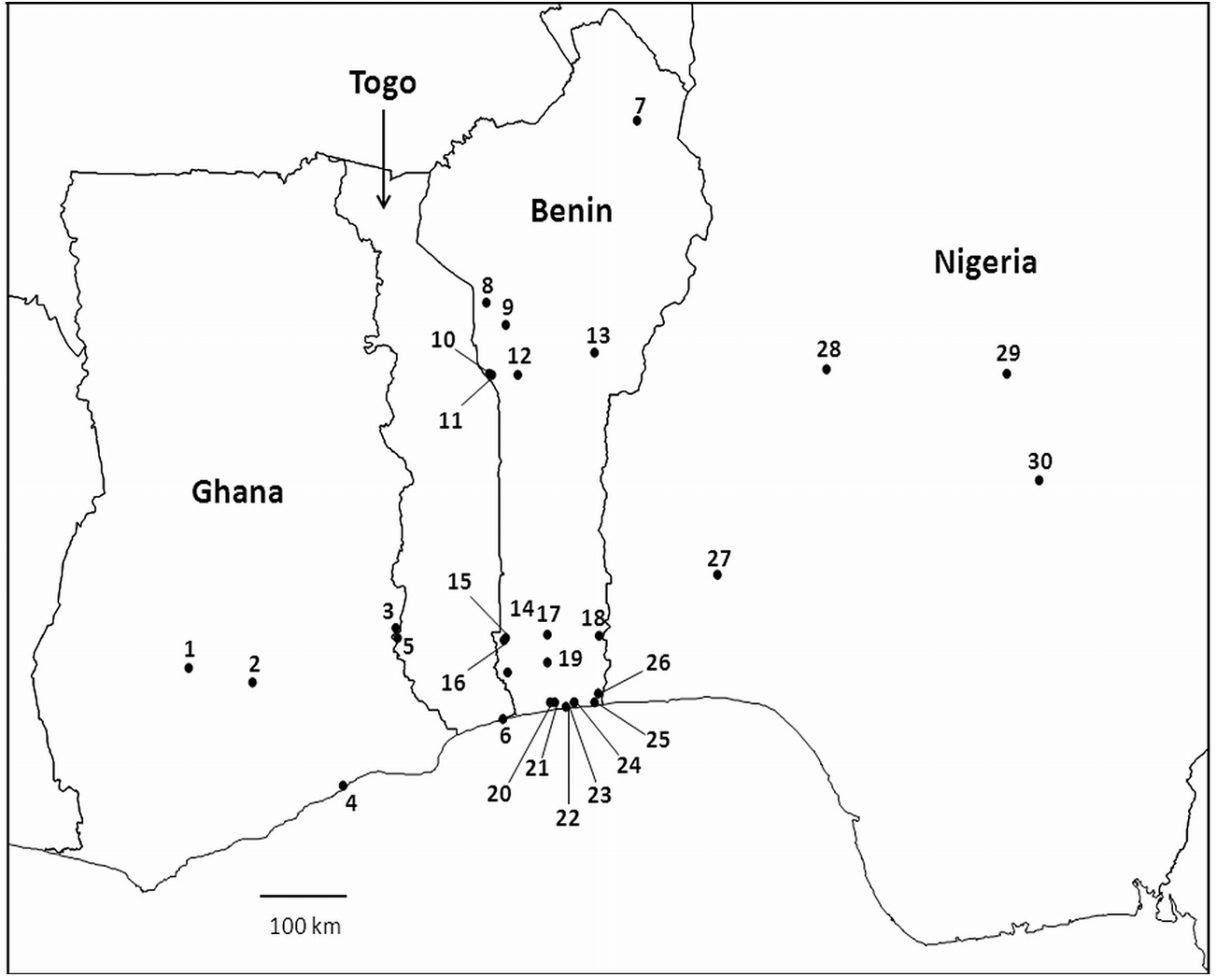
Map of the Central African region where hoverflies were collected (Ghana—Togo—Benin—Nigeria), showing the sampling localities (for more details see the S1 Table).

In order to address the relative paucity of available DNA barcodes for Afrotropical syrphids, we conducted a study of 98 nominal species, mainly from western Africa (Ghana, Togo, Benin, and Nigeria) (Fig 1; S1 Table) and constructed a DNA barcode library to identify hoverflies from this region. Multiple analytic methods were evaluated to get insight into the accuracy and shortcomings of the database. Finally, we illustrate how DNA barcoding can be used to complement and boost taxonomic research on this speciose insect group.

## Materials and Methods

### Specimens

A total of 640 hoverfly specimens were collected with sweep nets from different localities in Ghana, Togo, Benin and Nigeria, from May 1994 to December 2014 (Fig 1, S1 Table). Activities reported in the study were conducted neither in national parks nor in any protected areas where a special permit was required. Generally hover fly material was obtained from agricultural land and its adjacent environment. Private grounds were never penetrated without the consent of owners and were visited with national recruited staff and as part of the ongoing projects on pest control and biodiversity of the International Institute of Tropical Agriculture (IITA). IITA is a non-profit making international organisation and a member of the Consultative Group on International Agricultural Research Consortium deeply implicated in research-for-development work in collaboration with national partners of 25 countries in the humid and sub-humid zones of sub-Saharan Africa. Research work in Ghana, Benin, and Nigeria, where IITA is headquartered or has regional offices, is based on bilateral agreements in form of memorandums of understanding (MoU) signed by the ministries of agriculture of all respective governments (more information can be found on http://www.iita.org/). In these MoU's research work in the field is an integral part of IITA’s contracted mandate. In Togo, IITA maintains a close partnership with the National Plant Protection Service (SPV) and the university of Lomé through which material was obtained. Therefore, no specific permissions were required for the collected hoverfly material. None of the collected hoverfly species figure in any red list, are endangered, threatened or considered to be endangered in the involved countries. Similarly, no species collected in the present study are ranked in any IUCN list or protected by CITES.

Specimens collected before 2013 were pinned and stored in the dry collection at the International Institute for Tropical Agriculture (ITTA) in Calavi, Benin (N = 303); those collected in 2013–2014 were kept in absolute ethanol and are vouchered at the Royal Museum for Central Africa (RMCA, Tervuren, Belgium) (N = 337) (S1 Table). All specimens were identified based on external morphology by KJ and MDM (a list of the keys used is available in S1 Text), except for the Microdontinae which were identified by Menno Reemer (Naturalis Biodiversity Center, Leiden, The Netherlands). Whenever possible, identifications were checked against voucher specimens at the RMCA, the National Museum Bloemfontein (Bloemfontein, South-Africa), and the KwaZulu Natal Museum (Pietermaritzburg, South-Africa). Some specimens could not be unambiguously identified, but were linked to the morphologically most similar species by ‘cf.’ (*confer*) or the most similar genus by ‘sp.’. In both cases the specimens were treated as separate nominal species. One male *Syritta* specimen (voucher 422C03; labelled as *Syritta brevis/unicolor*, see S1 Table) keyed out at a position where Lyneborg & Barkemeyer [27], in their identification key on *Syritta*, noted “the unknown males of *S*. *unicolor* new species and *S*. *breva* new species will probably come out here” (i.e. the males of both species are unknown). As such we identified 101 nominal species from 28 genera. One leg of each specimen was preserved in 98% ethanol at -20°C for DNA sequencing.

### DNA extraction, amplification and sequencing

Genomic DNA was extracted using the NucleoSpin Tissue kit (Macherey-Nagel, Düren, Germany). After adding proteinase K, samples were incubated overnight at 56°C. A fragment of the 5’-end of COI was amplified using primer pair LCO1490 and HCO2198 [28]. Each PCR mixture (25 μl) contained 1x PCR buffer, 0.2 mM dNTPs, 0.4 μM of each primer, 2.0 mM MgCl_2_, 0.5 U of Taq DNA polymerase (Platinum, Invitrogen), 2 μl DNA extract and enough mQ-H_2_O to reach a total volume of 25 μl. The PCR protocol involved an initial denaturation period of 94°C for 4 min, followed by 35 cycles of 30 s at 94°C, 30 s at 45°C and 45 s at 72°C, and a final extension of 7 min at 72°C. Amplicons were cleaned using the NucleoSpin^®^ protocol (Macherey-Nagel, Düren, Germany) and bidirectionally sequenced (using the PCR primers) on an ABI 3130xl automated capillary DNA sequencer (Life Technologies) using the BigDye Terminator v.3.1. Cycle Sequencing Kit. Sequences were assembled in SeqScape v.2.5 (Applied Biosystems), and aligned, trimmed and translated into amino acid sequences in MEGA v.5.2 [29] to verify that they were free of stop codons and gaps (none were detected). All sequences were submitted to GenBank (Accession nos. KR830807- KR831281, KR632611-KR632615, and KT624201-KT624233). Additionally, we included the ten >550 bp DNA barcodes of nine nominal Syrphidae species from GenBank and BOLD which also occur in the Afrotropics (S2 Table).

### Data analysis

First, we described the success rate of obtaining DNA barcodes of >550 bp. This threshold was arbitrarily chosen to retain a high amount of barcodes without the loss of a long fragment of the barcode region in further analyses. Samples for which the PCR failed, or for which the sequencing yielded shorter fragments, were considered as unsuccessful. The success rate, i.e. the percentage of samples with barcodes >550 bp, of the pinned specimens was calculated for periods of five years (i.e., 1993–1997, 1998–2002, 2003–2007, and 2008–2012), and that for the ethanol stored material (2013–2014) was calculated separately. Then, three analytical approaches were employed to analyse the sequences and are briefly described below.

Kimura 2-parameter (K2P) [30] pairwise distances were calculated within and between species, genera, and subfamilies, since in DNA barcoding studies this is the most widely used distance measure, but see [31] for a discussion on the (inappropriate) use of this measure. Frequency distribution histograms of conspecific and heterospecific pairwise distances, for each of the three subfamilies and for each of the genera with >5 morphospecies, were constructed using the APE package 2.7–1 of R [32] to look for (the size of) barcode gaps [33]. If there is no overlap in the cumulative distribution curves of intra- and interspecific K2P distances, this was referred to as a ‘true barcoding gap’.

The proportion of correctly identified specimens was estimated using the Best Match (BM) and Best Close Match (BCM) criteria in the program SpeciesIdentifier [34]. According to BM, each query was assigned the species name of its best-matching sequence regardless of how similar the query and reference sequences were. Identification then was considered correct when both sequences were from the same species (true positive), incorrect if the query species differed from the closest reference species (false positive) or ambiguous if multiple species yielded a BM with the query species. BCM relies on a threshold value of sequence similarity. This threshold was determined as the ‘best compromise threshold’ based on cumulative distribution curves of intra- and interspecific K2P distances following [35]. The proportion of correct, ambiguous or incorrect identifications was calculated as above. Yet, with the latter method, queries that have no BCM below the threshold are discarded from the identifications (i.e. remained unidentified) (as true negative if identification was incorrect, as false negative if identification was correct). Note that species that are only represented by a single sequence in the dataset will generate incorrect identifications under the BM and BCM criteria, because there are no other conspecific reference sequences in the dataset with which they can match [36]. For this, we have eliminated species with only one barcode sequence (N = 32; S1 Table) from the BM and BCM analyses. In the presence of a true barcoding gap, identification success using BM and BCM is 100%.

A Neighbor-Joining (NJ) tree [37] (using K2P distances) and a maximum likelihood (ML) tree [38] were constructed after removing identical sequences with DAMBE v.5 [39]. We focused on whether individuals of the same species clustered together, rather than on the evolutionary relationships between species (i.e. tree-based identification sensu [13,14], see [34]). *Clistoabdominalis ancylus* (Pipunculidae, voucher JSS1353, GenBank accession no. DQ337639) was used as outgroup. The NJ-tree was constructed in MEGA v.5.2 [29] using 1,000 bootstrap replicates. In addition, the COI dataset was partitioned according to codon position and the Akaike Information criterion in jModelTest v.2 [38,40] was used to select the most appropriate model of evolution. These were the F81+I+G (first position), GTR+I+G (second position), and GTR+G (third position) model, respectively. Then, Garli v.2.01 [41] was used to perform a maximum likelihood (ML) analysis (two replicates; 200 bootstrap pseudoreplicates) taken into account the most appropriate models of evolution for each of the three codon positions.

## Results

**Fig 2:**
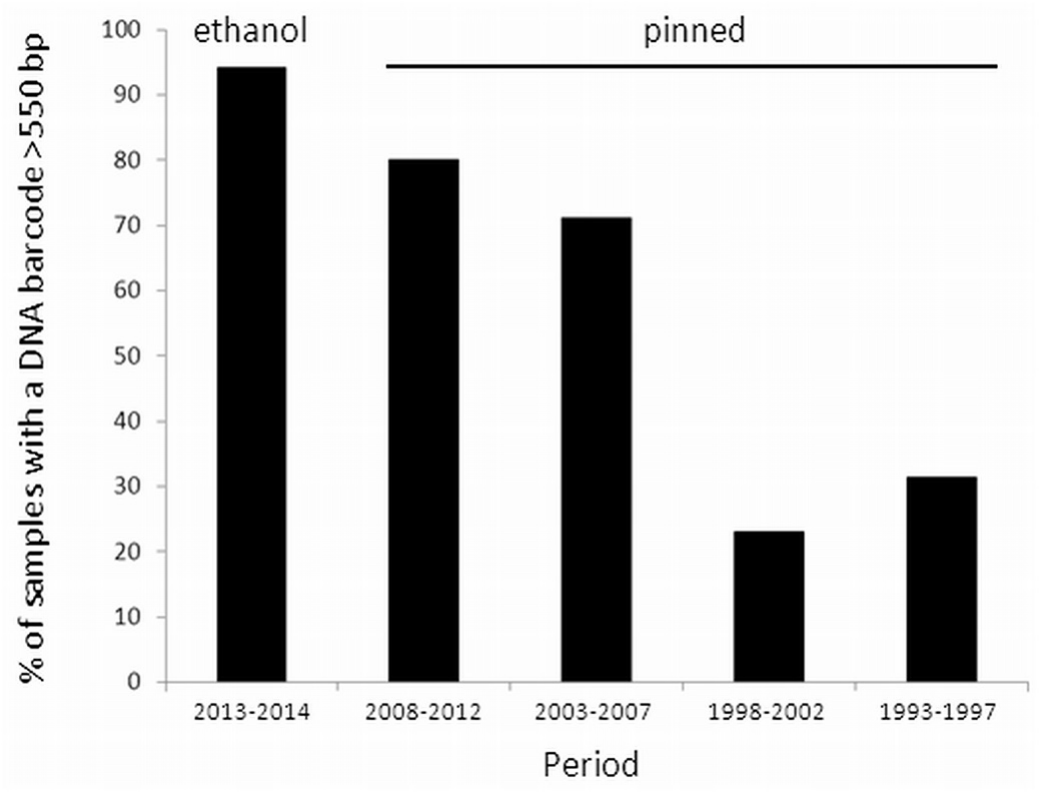
Frequency histogram of the percentage of samples for which a 550–657 bp COI barcode fragment was obtained. The left bar represents the ethanol preserved specimens (period 2013–2014), the other bars represent the pinned specimens (period 1993–2012).

A total of 513 out of the 640 individuals (80.2%) were successfully sequenced for a COI barcode fragment of >550 bp, representing 90 nominal species of 24 genera (Fig 1; S1 Table). For 11 nominal species no barcode was obtained. Likewise, for four genera (viz. *Ceratrichomyia*, *Meromacroides*, *Milesia*, and *Paramixogaster*) no barcodes could be sequenced successfully. The success rate of obtaining a >550 bp DNA barcode was higher for the recent, ethanol preserved specimens (321/337 or 95.3%), than for the older, pinned specimens (192/303 or 63.4%) of which the success rate dropped sharply for samples of >10 years old (Fig 2). Together with the ten >550 bp COI barcodes (from nine nominal species) from GenBank (S2 Table), the total DNA barcode dataset comprised 523 COI barcodes, from 98 nominal species belonging to 26 genera (S2 Text). More than one barcode was available for 66 of these taxa (S1 Table, S3 Table), while the remaining 32 had no conspecific in the dataset. One specimen of *Methadon* cf. *mythes* (voucher 414D05) clustered within a group of three *Microdon* (subgenus *Microdon*) specimens and not with two other *Methadon* cf. *mythes* individuals (S1 Fig). Since both genera are very divergent in morphology and DNA sequences it is most likely that this specimen was mislabeled and it was therefore discarded in the identification analyses.

**Fig 3:**
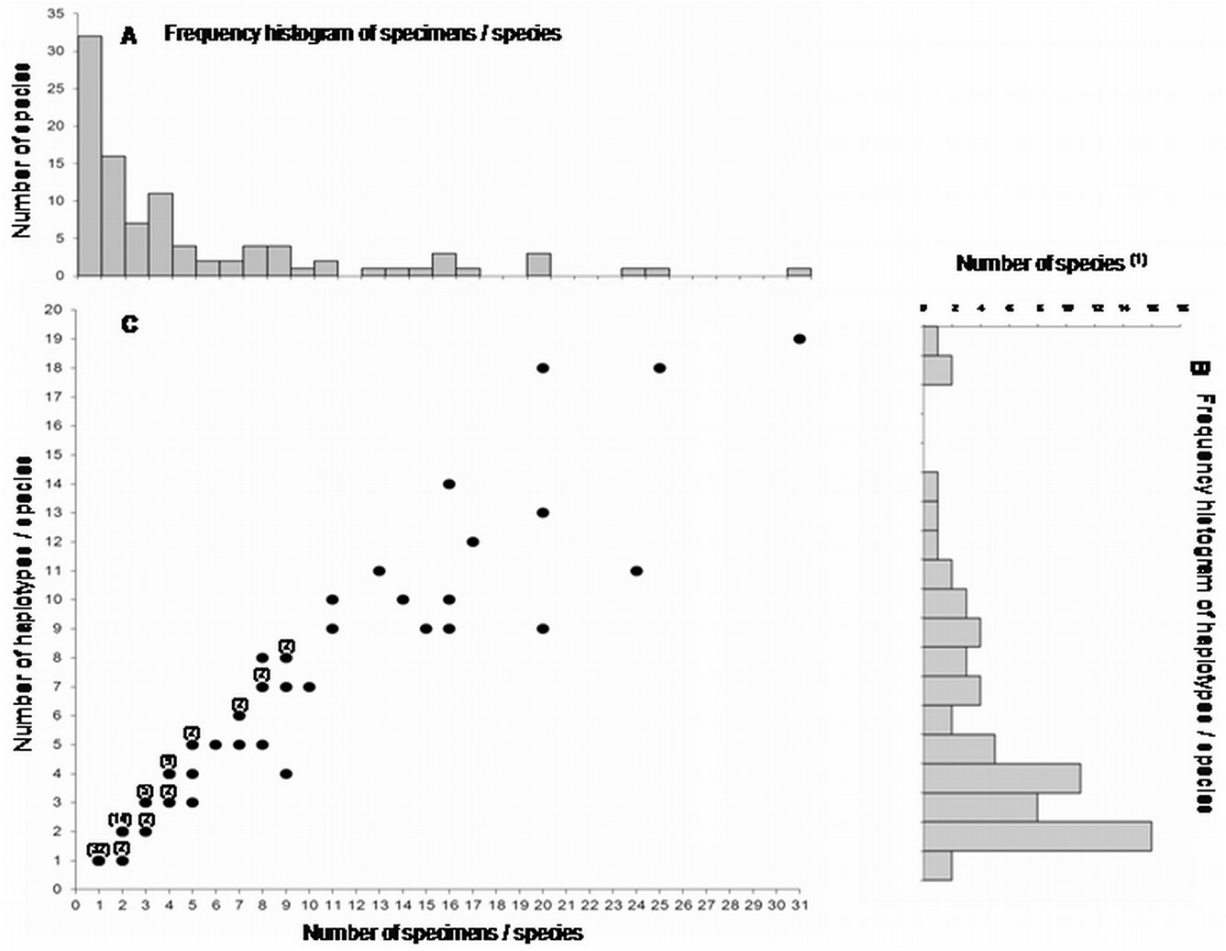
Frequency histograms of specimen numbers (A), number of haplotypes per species (B) and changes in the number of haplotypes with respect to the number of specimens sampled per species (C). In the latter, for each dot that represents more than one species, the number of species is given. ‘Species’ refers to the number of nominal species. (1): species represented by only one specimen are excluded.

We obtained a mean of 5.32 barcode sequences per species (4.13 if we only consider the unique haplotypes (i.e. individuals with similar barcodes are counted once), with 67.3% of the species represented by at least two barcodes (Fig 3A, S3 Table). If we excluded species represented by a single specimen (N = 32), the number of haplotypes per species (N = 66) ranged from 1 to 19 (Fig 3B, S3 Table), with a mean of 5.65 haplotypes per species. This mean number increased with the number of specimens sampled (Fig 3C). Haplotype numbers increased rapidly with the number of individuals sampled per species (Spearman Rank R_s_ = 0.971, t = 32.56, N = 66, *P* < 0.0001), but correlations with mean and maximum intraspecific K2P distances were less strong (R_s_ = 0.144, t = 1.17, N = 66, *P* = 0.12 and R_s_ = 0.51, t = 4.70, N = 66, *P* < 0.0001, respectively). Thus, greater intraspecific sampling yields more haplotypes, but has no major effect on intraspecific K2P distances.

The average K2P distances within species and among species within genus were 0.0073 (range 0–0.0563) and 0.0721 (range: 0–0.163), respectively. Of the 98 putative nominal species, 83 (84.7%) showed K2P distances of >0.03 from their nearest neighbor. Nine species pairs [15 nominal species (15.3%)] had a mean K2P distance <0.03 (Table 1), but no haplotypes were shared between species. The mean intraspecific K2P distance ranged from 0 to 0.0563 (S3 Table). High maximum intraspecific K2P divergences (>0.03) were observed for eight putative nominal species, viz. *Allobaccha picta*, *Asarkina ericetorum*, *Eristalinus vicarians*, *Graptomyza triangulifera*, *Phytomia natalensis*, *Polybiomyia divisa*, *Syritta bulbus*, and *S*. *lanipes* (S3 Table).

**Table 1:** Afrotropical hoverfly species pairs with small (< 0.03) mean interspecific K2P COI sequence distance.

Species pair	Mean	Min.	Max.
*Allobaccha euryptera*–*A*. *picta* (clade 1)	0.0051	0.0046	0.0061
*Allobaccha praeusta*–*A*. cf. *praeusta*	0.0030	0.0030	0.0030
*Asarkina ericetorum* (group 2)–*A*. *gemmata*	0.0009	0.0001	0.0061
*Eristalodes quinquelineatus*–*E*. *surcoufi*	0.0097	0.0016	0.0219
*Melanostoma bituberculatum*–*M*. cf. *floripeta*	0.0174	0.0198	0.0259
*Melanostoma* cf. *floripeta*–*M*. cf. *bituberculatum*	0.0295	0.0290	0.0305
*Melanostoma bituberculatum*–*M*. cf. *bituberculatum*	0.0169	0.0198	0.0245
*Microdon brevicornis*–*Archicrodon* sp.1	0.0284	0.0259	0.0306
*Rhingia caerulescens*–*R*. *semicaerulea*	0.0166	0.0166	0.0166

**Fig 4:**
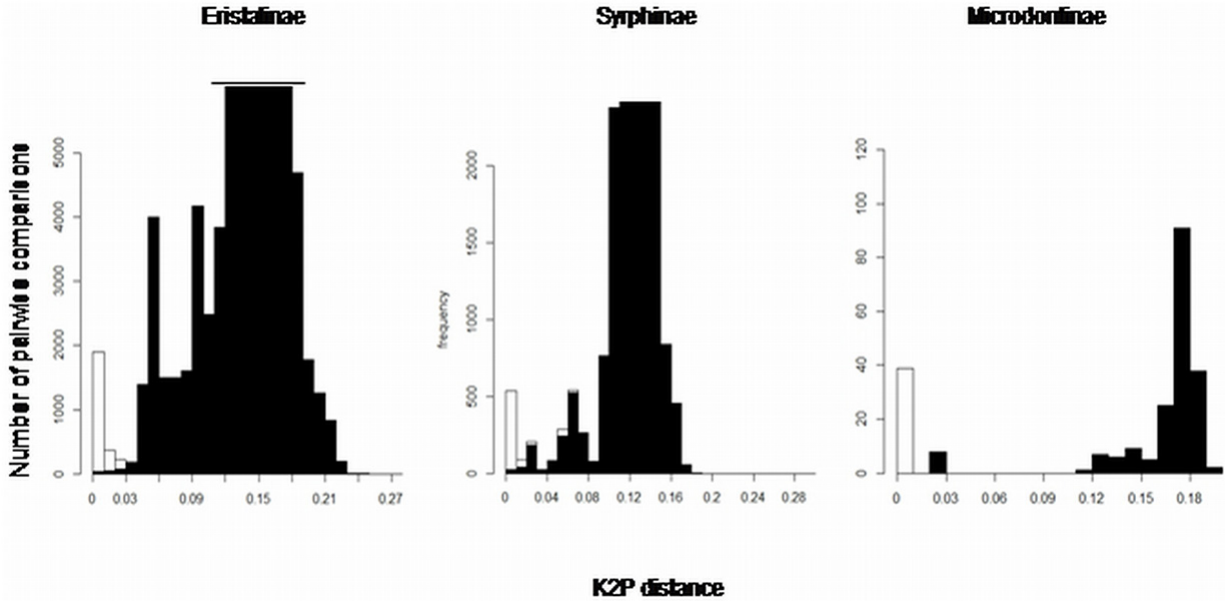
Frequency histogram of congeneric (open bars) and intraspecific (filled bars) Kimura 2-parameter (K2P) distances within three Central Afrotropical hoverfly subfamilies, based on COI barcodes.

The BM and BCM analyses were performed on 66 nominal species for which more than one barcode was available (Table 2; S3 Table). The best threshold distance to separate intra- from interspecific K2P distances was 0.057 for the entire dataset but with large differences in thresholds among the three subfamilies, viz. 0.037 (Eristalinae), 0.060 (Syrphinae), and 0.007–0.02 (Microdontinae) (Fig 4). Only for the Microdontinae there was a true barcode gap with no overlap between the frequency histograms of congeneric (minimal K2P distance: 0.02) and intraspecific (maximal K2P distance: 0.007) K2P distances. For all genera with >5 morphospecies, there was a true barcoding gap (subfamily Eristalinae: *Eristalinus*: 0.040–0.050, *Eumerus*: 0.011–0.099, *Mesembrius*: 0.017–0.040, *Phytomia*: 0.052–0.098, *Syritta*: 0.043; subfamily Syrphinae: *Paragus*: 0.013–0.067; subfamily Microdontinae: *Metadon*: 0.007–0.020), except for the genus *Allobaccha* (subfamily Syrphinae: optimal threshold: 0.053).

**Table 2:** Number of sequences (N seq.), number of putative nominal species (N sp.), and the number of putative nominal species for which no conspecific sequence was available in the dataset (N no con.) for the three subfamilies of Syrphidae, and the identification success according to the Best Match (BM) and Best Close Match (BCM) identification methods. TP = true positives, FP = false positives, FN = false negatives.

Subfamily	N seq.	N sp.	N no con.	Best Match (BM)			Best Close Match (BCM)			
				correct (TP)	ambiguous	incorrect (FP)	correct (TP)	ambiguous	incorrect (FP)	no match ˂ threshold (FN)
Microdontinae	22	8	3	19 (100%)	0 (0%)	0 (0%)	19 (100%)	0 (0%)	0 (0%)	0 (0%)
Eristalinae	350	57	18	332 (100%)	0 (0%)	0 (0%)	326 (98.2)	0 (0%)	0 (0%)	6 (1.8%)
Syrphinae	151	33	11	136 (97%)	0 (0%)	4 (2.9%)	136 (97.1%)	0 (0%)	4 (2.9%)	0 (0%)

Identification success for the three subfamilies is summarized in Table 2. For the Eristalinae, identification success for the BM method was 100%, that for the BCM method 98.2% with six individuals (1.8%) that had a closest match above the threshold, though the closest match was always a conspecific (Table 3). For the Syrphinae, identification success for the BM and BCM method was 97.1%. Four individuals (2.9%) were incorrectly identified, viz. three *Asarkina ericetorum* individuals were identified as *A*. *gemmata* while one *A*. *gemmata* individual was identified as *A*. *ericetorum* (Table 3). For the Microdontinae, identification success for the BM and BCM method was 100%. Likewise, for those genera with a true barcoding gap, identification success was 100%. Also for *Allobaccha*, BM and BCM identification success was 100% despite an overlap in the cumulative distribution curves of intra- and interspecific K2P distances. This is because the overlap is caused by morphospecies with single barcodes which are discarded in the calculation of BM and BCM identification success (see Materials and methods).

**Table 3:** Specific results for the Best Match (BM) and Best Close Match (BCM) identification methods (see also Table 2). The last column gives the optimal threshold used for the BCM method (see text and [35]).

Subfamily/Species	Voucher	BM/BCM	Voucher	K2P	Threshold
Correct identifications with BCM above optimal threshold within the Eristalinae (false negatives)					
*Polybiomyia divisa*	413E04	*P*. *divisa*	413C04	0.056	0.037
*Polybiomyia divisa*	413C04	*P*. *divisa*	413E04	0.056	0.037
*Phytomia natalensis*	417BO2	*Ph*. *natalensis*	417D02	0.043	0.037
*Graptomyza triangulifera*	413F01	*G*. *triangulifera*	413A02	0.041	0.037
*Syritta bulbus*	425D07	*S*. *bulbus*	JN992038	0.046	0.037
*Syritta bulbus*	JN992038	*S*. *bulbus*	425D07	0.046	0.037
Incorrect identifications (BM and BCM) for species within the Syrphinae (false positives)					
*Asarkina gemmata*	413D07	*A*. *ericetorum*	423B08	0.0001	0.060
*Asarkina ericetorum*	423E07	*A*. *gemmata*	413D07	0.0001	0.060
*Asarkina ericetorum*	423B08	*A*. *gemmata*	413D07	0.0001	0.060
*Asarkina ericetorum*	413B05	*A*. *gemmata*	413F06	0.048	0.060

Thirty-two of the 98 nominal species (32.7%) for which we obtained a barcode(s) were represented by a single branch (i.e. single haplotype) in the NJ/ML-tree (S1 Fig). Of the 66 nominal species with ≥2 haplotypes, 60 (91%) formed clusters with high (>98%) bootstrap support in the NJ-analysis, while bootstrap support for the clusters was generally lower in the ML-analysis (32 morphospecies (48%) with bootstrap support >98%). Two nominal species (3%) formed clusters with low (<70%) bootstrap support in the NJ/ML-analysis, viz. *Allobaccha* cf. *praeusta* and *Melanostoma bituberculatum*, while in the ML-analysis also the clusters of *Eristalodes quinquelineatus* and *Melanostoma* cf. *bituberculatum* had low (< 70%) bootstrap support. The barcodes of four nominal species (6%) did not form single clusters. The barcodes of *Allobaccha picta* formed two clusters and one of these included the single barcode of *A*. *euryptera*. The barcodes of *Asarkina ericetorum* formed a cluster with the barcode of *Asarkina* sp. 1 and those of *A*. *gemmata* which neither formed a single cluster. Finally, the single barcode of *Eristalodes surcoufi* was nested within the cluster of *Eristalodes quinquelineatus* barcodes.

## Discussion

### Restrictions to the current DNA barcode reference dataset

We evaluated the use of DNA barcoding to identify 90 Afrotropical hoverfly species collected in an area that largely corresponds to the Dahomey Gap, a West African region where the Guinean Forest-Savanna Mosaic reaches the coastal area, thereby splitting the coastal forest ecoregions (with the Eastern Guinean Forests ecosystem to the West, and the Nigerian Lowland Forests ecosystem to the East) [42]. It is currently composed of a mosaic of savannahs, plantations, degraded forest and remnants of natural forest. Public DNA sequence libraries (GenBank and BOLD) lack representative reference barcodes for these, and almost all other, Afrotropical Syrphidae even though about 600 species currently have been described [1,7,8,9].

One reason for the lack of DNA barcodes of Afrotropical hoverflies in public databases is that much of the material is pinned and old. Indeed, the success rate of obtaining a barcode sharply dropped for samples of >10 years old (20% success rate), a phenomenon that is common among pinned insects [43,44]. Nested PCR and mini-barcode approaches may overcome the low PCR and DNA sequencing success rate of old, pinned specimens e.g. [45,46], but are more time-consuming and labor intensive, especially if new primers need to be developed. Next-generation DNA barcoding may become a better solution because of its protocol simplicity, reduced cost per barcode read, faster throughout, and added information content [47]. Currently, using Sanger-sequencing our database represents roughly one third of the more common species (estimated from records given by [48]), and does not include any type-specimen.

Obviously, our dataset does not span the entire taxonomic diversity of African hoverflies and two-thirds of the more common species, and representatives from more than 20 genera, are not represented in the dataset (because of the restricted sampling area). Besides the lack of many species in the dataset, we found a highly significant correlation between the number of individuals sampled per species and the number of haplotypes recovered, and between the number of species sampled per genus and the number of known species in Africa, even though the mean, and maximum, K2P distances were not, or less, affected by sample size. Incorrect species assignments in Afrotropical Syrphidae thus may be because of errors in the construction of the barcode database. This problem is inherently associated with barcoding studies of unknown biotas, such as Afrotropical hoverflies for which only approximately 60% of the known species can be identified using the current identification keys [8]. The more speciose genera even pose more of a problem, because keys for these genera cover only 44% of the Afrotropical fauna [8]. For instance, the genus *Eumerus* is probably the most speciose hoverfly genus in the Afrotropics with more than 70 species [48] but current identification keys have many shortcomings and species descriptions of many species are vague, insufficient, or based on a single sex. Nevertheless, DNA barcode studies such as ours may set a reference, and may improve or boost ongoing taxonomic studies on the Afrotropical Syrphidae. Conducting similar studies for representative collections of other geographical parts or biomes of the continent may rapidly increase our taxon coverage (including generic level), and thereby increasing the value of the DNA barcode database as a useful tool for identification and other research purposes.

Despite of the shortcomings listed above, DNA barcoding works well for most of the nominal species we examined, and various analytical methods give highly congruent results. In general, the mean intraspecific divergence of 0.0078 (range 0–0.0563) is at the lower end of the distribution of intraspecific divergences found in other insect groups, while the mean interspecific divergence of 0.093 (range 0–0.163) is relatively high for congeneric insect species [49]. Yet, the interspecific divergence distribution overlaps the intraspecific divergence distribution in the subfamilies Syrphinae and Eristalinae, resulting in the absence of a perfect DNA barcoding gap [50]. This makes it impossible to define a general distance threshold for hoverflies. However, some methods allow to estimate an *ad hoc* threshold for a specific reference dataset so that assignment errors can be minimized e.g. [36]. Such threshold was lower for the Eristalinae (0.037) than for the Syrphinae (0.06), and were much higher than for the Microdontinae that showed a true barcoding gap (0.007–0.02) (i.e. there is no overlap in the frequency histograms of congeneric and intraspecific K2P-distances; see Fig 4C). Our results further show that optimal thresholds may differ strongly among genera and thus is seems more appropriate to calculate optimal thresholds at the genus level to discriminate between intra- and interspecific K2P distances. In our dataset, incorrect assignments were caused both by high intraspecific, and low interspecific, sequence divergence, and this suggests that the taxonomy of several taxa deserves further study, or, alternatively, that DNA barcoding may not work for some genera or species groups.

### Taxonomic implications

Meier et al. [34] investigated whether DNA barcodes could be used for species identification in 449 species (1333 barcodes) of Diptera and found a relatively low success rate (< 70%) based on tree-based and other proposed species identification criteria. Yet, DNA barcoding allows the fast detection of shallow interspecific, or deep intraspecific, barcode divergences and may facilitate the selection of taxa for future taxonomic work [15,19]. As such, we can depict problematic taxa, or species groups, within the Afrotropical Syrphidae that warrant further taxonomic study. Low interspecific differentiation was observed in five nominal species pairs, viz. *Allobaccha euryptera*—*A*. *picta*, *Allobaccha praeusta*—*A*. *cf*. *praeusta*, *Asarkina ericetorum*—*A*. *gemmata*, *Eristalinus quinquelineatus*—*A*. *surcoufi*, *Microdon brevicornis*–*Archmicrodon* sp.1, *Rhingia caerulescens*–*R*. *semicaerulea*, and *Melanostoma bituberculatum*–*M*. cf. *floripeta*–*M*. cf. *bituberculatum*, whereas high intraspecific differentiation was observed in *Allobaccha picta*, *Asarkina ericetorum*, *Syritta bulbus*, *Phytomia natalensis*, *Eristalodes quinquelineatus*, *Eristalinus vicarians*, *Graptomyza triangulifera*, and *Polybiomyia divisa*. Such observations may reflect geographical structuring or evolutionary history. Some of these lineages may represent recently diverged species in which the COI sequences has not yet accumulated many mutations or may represent ancestral polymorphisms that have been retained in the two taxa. This may not be very surprising for a group that has received little taxonomic attention. One obvious genus that illustrates well how DNA barcoding may be an onset for a taxonomic revision is *Allobaccha* for which we observed both low inter- and high intraspecific sequence divergence, and a number of putative nominal species, which we could not identify using the current literature/keys, and that may represent undescribed taxa. Perhaps this is even better illustrated by the Microdontinae of our dataset where none of the individuals for which we obtained a COI barcode currently could be attributed to a known species (see S1 Table). Two *Metadon* taxa, viz. *Metadon* cf. *mythes* and *M*. cf. *inermis*, morphologically closely resemble known species, and five other clades in the NJ-tree seem to represent undescribed species. Also the *Paramixogaster* specimen for which we could not obtain a COI barcode, seems to be an undescribed taxon (Menno Reemer, personal communication). Obviously, there is a need for more sequence data and the study of more variable DNA markers to improve resolution but also of a re-evaluation of the morphological characters that currently are used to separate the nominal species (see also [51]). Only such an integrative approach will allow to highlight the effects for the lack of correspondence between sequence variants and current nominal species, in a similar way as integrated taxonomy has improved the taxonomy of the *Merodon equestris* species complex [52].

### Identification of introduced species

DNA barcoding allowed to detect an introduced hoverfly species in the Afrotropics, viz. *Toxomerus floralis* (Fabricius, 1798). Obviously, the specimens could not be identified using the current literature and identification keys on Afrotropical Syrphidae. Using the search engines BOLD Identification System (BOLD-IDS) and GenBank's BLAST the specimens were assigned to *T*. *floralis*. A subsequent morphological identification using specific keys for *Toxomerus* confirmed the species’ identification [53]. This New Word species is only the second New World species that has been introduced into the Old World and it seems well-established in Togo, Benin, Nigeria and Cameroon. Hence, DNA barcoding may not only facilitate the discovery and identification of (recent) introductions but it may also allow a better bio-monitoring of the species and of the potential impact on endemic plant-pollinator communities and ecosystems [54].

### Linking sexes and life history stages of species

The larvae of hoverflies show a variety of feeding modes. Information on the feeding mode, and associated feeding morphology, may yield important information to understand the evolutionary and phylogenetic relationships of hoverflies, since morphological innovation is often associated with feeding modes [55,56]. Unfortunately, such information is lacking for most hoverfly species. For instance, food plants (oviposition sites) are known, and descriptions of immature stages have been provided, for <8% of known phytophagous hoverflies [55,56]. Andrić et al. [57] showed that DNA barcoding is a useful tool to identify the larvae of *Merodon* species. Similarly, our DNA barcoding reference library of Afrotropical hoverflies may enhance our understanding of the (feeding) ecology and morphology for Afrotropical hoverfly species. In the same view, DNA barcoding will be highly valuable in linking sexes of species, especially since perhaps for even more than 30% of the current Afrotropical hoverfly species only one sex is known. For instance, previously supposed females of *Mesembrius ingratus* (Loew, 1858) were all attributed to *Mesembrius tarsatus* (Bigot, 1883) [58] so that the female of *M*. *ingratus* remained unknown. We here show that males of both species have a very low intraspecific sequence divergence (*M*. *ingratus*: 0.0022, *M*. *tarsatus*: 0.0015), yet a high mean K2P-sequence divergence of 0.09. Two females (vouchers 106E08 and 417E08) clustered within the males of *M*. *ingratus* while seven other females (vouchers 106E02, 106E04, 107A08, 107C03, 425F06, 418B03, 418C03) clustered within the males of *M*. *tarsatus*. We thus have, for the first time, individuals that can be unambiguously identified as females of *M*. *ingratus*. This result will further allow to examine the females of both species for morphological diagnostic characters, and to improve the morphological identification key for the genus *Mesembrius* (Jordaens, Goergen, Backeljau & De Meyer, unpublished data). Another example is the one male *Syritta* specimen (voucher 426A01) that in the identification key of [27] keyed out at a position where the unknown males of *Syritta brevis* and *S*. *unicolor* are supposed to key out. Unfortunately, the reference database does not contain barcodes of females of both species so that the identification of the male specimen will not be unambiguous.

## Conclusions

We here provide the first COI barcode reference database for approximately one third of the more common Afrotropical hoverfly species. The reference database will not only assist in identifications, but also provides a basis to pinpoint taxa that need further taxonomic study, helps to identify recent introductions, and can be used to link sexes, and larvae with adults, of a species. However, the database should be expanded since still many Afrotropical species and genera are missing. Ideally, the database should also include specimens from a larger geographic area, and other ecoregions, to account for intraspecific variation in barcodes. Such a large-scale DNA barcoding study of the Afrotropical Syrphidae is currently ongoing (Jordaens, Goergen, Muller, Kirk-Spriggs, Backeljau, De Meyer et al., unpublished data).

## Supporting Information

S1 Fig Neighbor-Joining tree (K2P distances) of COI made with 523 Afrotropical hoverflies from 98 nominal species (26 genera). Bootstrap support values >70% are shown at the nodes as: Neighbor-Joining / Maximum Likelihood.
(PDF)

S1 Table List of Afrotropical hoverfly species (Syrphidae) used in this study. (XLSX)

S2 Table List of COI barcodes for Afrotropical hoverfly species from GenBank or publically available in BOLD. Accessions in bold are those with a fragment size >550 bp and were include in the analyses. Note that the accessions for *Paragus tibialis* are from European specimens. The species, however, also occurs in the Afrotropics and we have barcodes of congenerics with whom it could be misidentified.
(DOCX)

S3 Table Summary of the intraspecific percentage genetic divergences (K2P model) of 66 Afrotropical putative hoverfly species and the number of COI barcodes (No. barcodes), and unique COI barcodes (No. unique barcodes). The 32 putative nominal species that were only represented by one specimen are not shown. Values of the maximum K2P intraspecific distance >0.05 are in bold and underlined.
(DOCX)

S1 Text Overview of the literature with morphological identification keys, and other relevant literature, used to identify the Afrotropical hoverflies of this study. (DOCX)

S2 Text Alignment of a 550–657 bp region of the cytochrome *c* oxidase subunit I (COI) of 523 Afrotropical Syrphidae from 98 putative nominal species of 26 genera, and *Clistoabdominalis ancylus* (Pipunculidae; outgroup) used in this study. (TXT)
