# Supplementary figures and images for: DNA Barcoding to Improve the Taxonomy of the Afrotropical Hoverflies (Insecta: Diptera: Syrphidae)
Source: PLoS One. 2015 Oct 16;10(10):e0140264. doi: 10.1371/journal.pone.0140264 (PMC4608823; doi:10.1371/journal.pone.0140264)

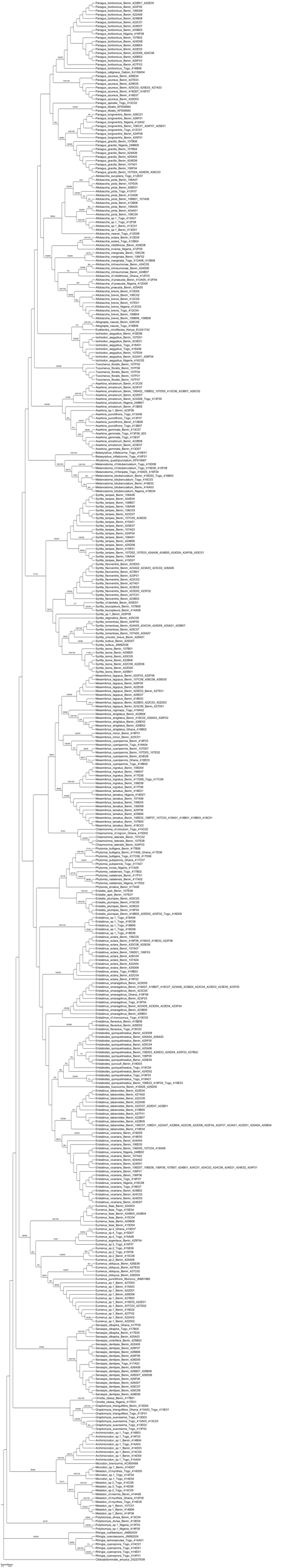

Supplement: S1 Fig — Bootstrap support values >70% are shown at the nodes as: Neighbor-Joining / Maximum Likelihood. (PDF) [file pone.0140264.s001.pdf]
