# Supplementary material for: DNA Barcoding to Improve the Taxonomy of the Afrotropical Hoverflies (Insecta: Diptera: Syrphidae)
Source: PLoS One. 2015 Oct 16;10(10):e0140264. doi: 10.1371/journal.pone.0140264 (PMC4608823; doi:10.1371/journal.pone.0140264)
Supplement: S2 Table — Accessions in bold are those with a fragment size >550 bp and were include in the analyses. Note that the accessions for Paragus tibialis are from European specimens. The species, however, also occurs in the Afrotropics and we have barcodes of congenerics with whom it could be misidentified. (DOCX) [file pone.0140264.s003.docx]

**Supporting Information Table S2** List of COI sequences (barcodes) for Afrotropical hoverfly species from GenBank or publically available in BOLD. Accessions in bold are those with a fragment size >550 bp and were include in the analyses (see text for more explanation). Note that the accessions for *Paragus tibialis* are from European specimens. The species, however, also occurs in the Afrotropics and because barcodes from other Afrotropical *Paragus* were obtained, the species was included in the analyses.

Species voucher/strain GenBank length locality

accession no. (bp)

***Afrostoma quadripunctatum* USNM ENT 36401 KF919067 647 Kenya**

*Allograpta calopus* MZH:XP39 EF127311 0 South Africa

*Allograpta fuscotibialis* MZH:XP37 EF127309 0 South Africa

*Asarkina ericetorum* MZH:S222 EF127353 396 Kenya

*Asarkina fulva* XP100 EU241738 427 Madagascar

*Episyrphus stuckenbergi* MZH:XP52 EF127319 396 Madagascar

*Episyrphus trisectus* CNC;Diptera:105397 KF026758 307 Uganda

*Episyrphus trisectus* CNC;Diptera:105398 KF026748 307 Uganda

*Episyrphus trisectus* CNC;Diptera:105399 KF026751 307 Uganda

*Episyrphus trisectus* CNC;Diptera:105400 JN991981 307 Uganda

***Eumerus punctifrons* CNC:Diptera:102104 JN991989 657 Morocco**

***Exallandra cinctifacies* MZH:XP148 EU241742 643 Kenya**

*Ischiodon aegyptius* S361 AY603769 396 Morocco

*Ischiodon aegyptius* MZH:XP48 EF127312 0 Madagascar

*Melanostoma annulipes* MZH:XP53 EF127320 396 Madagascar

***Microdon brevicornis* CNC:Diptera:102744 KC900499 657 South Africa**

*Microdon clatratus* MZH:Y778 HF936693 396 Madagascar

*Paragus borbonicus* S220 AY476850 396 Kenya

***Paragus caligneus* ZFMK D067 KJ158454 657 Gabon**

*Paragus haemorrhous* S115Phae AY174471 396 South Africa

*Paragus longiventris* S284 AY476859 0 Tanzania

*Paragus* aff. *manensis* S248 AY476860 396 ‘Africa’

*Paragus pusillus* S300 AY476861 0 Madagascar

***Paragus tibialis* PAR-TIB-1 KF939564 580 Spain**

***Paragus tibialis* PAR-TIB-2 KF939565 603 Spain**

*Paragus tibialis* PTIBIS52 AY174465 396 Spain

*Paragus tibialis* S326 AY476841 396 Finland

*Paragus tibialis* S96Ptibi AY174468 396 Greece

***Rhingia coerulescens* CNC:Diptera:102556 JN992024 657 Uganda**

***Rhingia cuthbertsoni* CNC:Diptera:102559 JN992025 657 Kenya**

*Rhingia trivittata* MZH:G47 AY533316 396 Kenya

***Syritta bulbus* CNC:Diptera:102674 JN992038 657 Uganda**
