# Supplementary material for: DNA Barcoding to Improve the Taxonomy of the Afrotropical Hoverflies (Insecta: Diptera: Syrphidae)
Source: PLoS One. 2015 Oct 16;10(10):e0140264. doi: 10.1371/journal.pone.0140264 (PMC4608823; doi:10.1371/journal.pone.0140264)
Supplement: S3 Table — The 32 putative nominal species that were only represented by one specimen are not shown. Values of the maximum K2P intraspecific distance >0.05 are in bold and underlined. (DOCX) [file pone.0140264.s004.docx]

**Supporting Information Table S3** Summary of the intraspecific genetic divergences (K2P model) of 66 Afrotropical putative hoverfly species and the number of COI barcodes (No. barcodes), and unique COI barcodes (No. unique barcodes). The 32 putative morphospecies that were only represented by one specimen are not shown. Values of the maximum K2P intraspecific distance >0.05 are in bold and underlined.

Species Mean S.E. Min Max No. barcodes No. unique

barcodes

*Allobaccha brevis* 0.0067 0.0019 0 0.0122 9 8

*Allobaccha* cf. *praeusta* 0.0020 0.0014 0 0.0030 3 2

*Allobaccha eclara* 0.0111 0.0042 0.0111 0.0111 2 2

*Allobaccha ichneumonae* 0.0041 0.0019 0.0030 0.0046 3 3

*Allobaccha marginata* 0.0160 0.0037 0 0.0213 4 3

*Allobaccha picta* 0.0307 0.0043 0 **0.0609** 11 10

*Allobaccha* sp.1 0.0048 0.0019 0.0015 0.0082 4 4

*Allograpta nasuta* 0.0046 0.0025 0.0046 0.0046 2 2

*Asarkina ericetorum* 0.0263 0.0033 0 **0.0670** 15 8

*Asarkina gemmata* 0.0037 0.0018 0 0.0061 4 4

*Asarkina punctifrons* 0.0023 0.0012 0.0015 0.0030 4 4

*Betasyrphus inflaticornis* 0.0015 0.0014 0.0015 0.0015 2 2

*Chasmomma lateralis* 0.0041 0.0019 0.0015 0.0076 3 3

*Eristalinus eclara* 0.0020 0.0007 0 0.0076 17 14

*Eristalinus flaveolus* 0.0021 0.0015 0.0016 0.0032 3 3

*Eristalinus smaragdinus* 0.0022 0.0007 0 0.0065 20 9

*Eristalinus tabanoides* 0.0031 0.0010 0.0000 0.0192 24 11

*Eristalinus vicarians* 0.0081 0.0013 0 **0.0396** 31 19

*Eristalinus* sp.1 0.0076 0.0024 0.0015 0.0141 5 5

*Eristalis apis* 0.0076 0.0031 0.0076 0.0076 2 2

*Eristalis plumipes* 0.0029 0.0010 0.0000 0.0091 8 5

*Eristalodes fuscicornis* 0 0 0 0 2 2

*Eristalodes quinquelineatus* 0.0134 0.0026 0 **0.0305** 20 13

*Eumerus feae* 0.0026 0.0011 0 0.0049 7 6

*Eumerus obliquus* 0.0054 0.0020 0.0031 0.0077 4 4

*Eumerus* sp. 1 0.0032 0.0011 0 0.0086 13 11

*Eumerus* sp. 2 0.0095 0.0027 0.0070 0.0117 4 4

*Eumerus* sp. 4 0.0031 0.0018 0.0015 0.0046 3 3

*Graptomyza suavissima* 0.0014 0.0009 0.0000 0.0035 5 5

*Graptomyza triangulifera* 0.0213 0.0037 0 **0.0426** 4 3

*Ischiodon aegyptius* 0.0021 0.0011 0 0.0061 9 8

*Melanostoma bituberculatum* 0.0088 0.0027 0 0.0152 6 5

*Melanostoma* cf. *floripeta* 0 0 0 0 2 1

*Melanostoma* cf. *bituberuculatum* 0.0010 0.0010 0 0.0015 3 2

*Mesembrius cyanipennis* 0.0100 0.0024 0 0.0183 8 7

*Mesembrius ingratus* 0.0022 000103 0 0.0047 7 6

*Mesembrius lagopus* 0.0055 0.0013 0 0.0139 16 9

*Mesembrius minor* 0.0076 0.0033 0.0076 0.0076 2 2

*Mesembrius strigilatus* 0.0018 0.0008 0 0.0046 7 5

*Mesembrius tarsatus* 0.0014 0.0005 0 0.0065 16 10

*Metadon* sp.1 0.0054 0.0023 0.0015 0.0081 3 3

**Supporting Information Table S4 (continued)**

*Metadon* cf. *mynthes* 0.0063 0.0028 0.0063 0.0063 2 2

*Metadon* sp.3 0.0066 0.0031 0.0066 0.0066 2 2

*Microdon* sp.1 0.0008 0.0007 0 0.0016 4 4

*Archimicrodon* sp.1 0.0044 0.0014 0.0016 0.0081 8 8

*Ornidia obesa* 0.0015 0.0014 0.0015 0.0015 2 2

*Paragus azureus* 0.0021 0.0008 0 0.0061 10 7

*Paragus borbonicus* 0.0072 0.0016 0 0.0158 20 18

*Paragus gracilis* 0.0041 0.0011 0 0.0111 11 9

*Paragus longiventris* 0.0026 0.0009 0 0.0076 9 7

*Paragus tibealis* 0 0 0 0 2 2

*Phytomia bulligera* 0.0015 0.0010 0 0.0030 5 3

*Phytomia natalensis* 0.0293 0.0044 0.0046 **0.0505** 4 4

*Phytomia pubipennis* 0.0076 0.0032 0.0076 0.0076 2 2

*Polybiomyia divisa* 0.0563 0.0088 0.0563 **0.0563** 2 2

*Polybiomyia* sp.1 0.0018 0.0017 0.0018 0.0018 2 2

*Rhingia cyanoprora* 0.0068 0.0025 0 0.0098 4 4

*Senaspis dentipes* 0.0053 0.0014 0 0.0184 16 14

*Senaspis dibapha* 0.0023 0.0014 0.0015 0.0030 4 4

*Syritta bulbus* 0.0457 0.0080 0.0457 **0.0457** 2 2

*Syritta flaviventris* 0.0049 0.0012 0 0.0122 14 10

*Syritta lanipes* 0.0144 0.0025 0 **0.0304** 25 18

*Syritta leona* 0.0040 0.0015 0 0.0091 8 7

*Syritta leucopleura* 0.0032 0.0022 0.0032 0.0032 2 2

*Syritta tomentosa* 0.0016 0.0008 0 0.0046 9 4

*Toxomerus floralis* 0.0052 0.0018 0.0016 0.0088 5 5
