# Supplementary material for: DNA Barcoding to Improve the Taxonomy of the Afrotropical Hoverflies (Insecta: Diptera: Syrphidae)
Source: PLoS One. 2015 Oct 16;10(10):e0140264. doi: 10.1371/journal.pone.0140264 (PMC4608823; doi:10.1371/journal.pone.0140264)
Supplement: S1 Text — (DOCX) [file pone.0140264.s005.docx]

**S3 Table List of identification keys and other relevant literature used to identify the Afrotropical hoverflies of this study.**

Note 1: Identifications to the genus level was done with an unpublished key to the genera of Afrotropical Syrphidae that was originally composed by Christian F. Thompson. An updated version of the key with illustrations will be published as a forthcoming chapter of the Manual of Afrotropical Diptera as Ssymank A, De Meyer M, Reemer M, Rotheray GE (submitted) 60. Syrphidae. In: Kirk-Spriggs AH, Sinclair BJ (Eds). Manual of Aftrotropical Diptera. Volume 2. Brachycera-Cyclorrhapha, excluding Calyptratae. Suricata XX. Pretoria: SANBI Publishing.

Note 2: References for the different genera of Microdontinae have been included, and are listed under the genus *Microdon*; note that specimens of this subfamily were kindly identified by Menno Reemer.

Note 3: some genera are only represented by single species in the Afrotropics (e.g. *Meromacroides*, *Milesia*, *Ischiodon*, *Ornidia*) and no keys are available; these genera are therefore not listed below. These specimens were identified with the key to the genera of Aftrotropical Syrphidae (see Note 1) and the original species descriptions.

Note 4: The references below are grouped per genus and include publications with keys and other relevant literature that was consulted (e.g. publications that contained additional species descriptions, comparisons with congeneric species, and/or helpful illustrations). Publication providing an identification key were indicated with “(key)” at the end of the reference.

Note 5: Additional useful references can be found in Whittington AE (2003) The Afrotropical Syrphidae fauna: an assessment. Studia dipterologica 10: 579-607.

***Allobaccha*:**

Bezzi M (1915) Syrphidae of the Ethiopian region based on material in the collection of the British Museum (Natural History), with descriptions of new genera and species. London, Trustees of the British Museum, pp.38-50. (key)

Curran CH (1927) Article II. – Diptera of the American Museum Congo Expedition. Part I. – Bibionidae, Bombyliidae, Dolichopodidae, Syrphidae and Tryaneidae. Bulletin of the American Museum of Natural History 57: 33-89. (key)

Curran CH (1938) Records and descriptions of African Syrphidae- II (Diptera). American Museum Novitates 1010: 1-20. (key)

Dirickx HG (2010) Notes sur le genre *Allobaccha* Curran, 1928 (Diptera, Syrphidae) à Madagascar avec description de cinq nouvelles espèces. Revue Suisse de Zoologie 117 : 213-233.

Kassebeer CF (2000) Zur Gattung *Pseudodoros* Becker, 1903 (Diptera, Syrphidae). Dipteron 3: 73-92.

***Allograpta*:**

Bezzi M (1915) Syrphidae of the Ethiopian region based on material in the collection of the British Museum (Natural History), with descriptions of new genera and species. London, Trustees of the British Museum, pp.36-38. (key)

Curran CH (1938) Records and descriptions of African Syrphidae- II (Diptera). American Museum Novitates 1010: 1-20. (key)

Kassebeer CF (2000) Die Schwebfliegen (Diptera, Syrphidae) von La Réunion, mit Anmerkungen zur Fauna der Madagassis. Dipteron 3: 43-66.

***Archimicrodon:*** (see *Microdon*). Identified by Menno Reemer

***Asarkina*:**

Bezzi M (1908) Secundo contributo alla conoscenza del genere *Asarcina*. Annales Historico-Naturales Musei Nationalis Hungarici 6: 495-504. (key)

Bezzi M (1912) Ditteri raccolti da Leonardo Fea durante il suo viaggio dell’Africa occidentale. Parte Ia: Syrphidae. Annali del Museo Civico di Storia Naturale di Genova 5: 400-443. (key)

Bezzi M (1915) Syrphidae of the Ethiopian region based on material in the collection of the British Museum (Natural History), with descriptions of new genera and species. London, Trustees of the British Museum, pp.21-29. (key)

Curran CH (1927) Article II. – Diptera of the American Museum Congo Expedition. Part I. – Bibionidae, Bombyliidae, Dolichopodidae, Syrphidae and Tryaneidae. Bulletin of the American Museum of Natural History 57: 33-89. (key)

Curran CH (1938) Records and descriptions of African Syrphidae- II (Diptera). American Museum Novitates 1010: 1-20. (key)

Keiser F (1971) Syrphidae von Madagascar (Dipt.). Verhandlungen der Naturforschenden Gesellschaft in Basel 81: 223-318.

Vockeroth JR (1969) A revision of the genera of the Syrphini (Diptera: Syrphidae). Memoirs of the Entomological Society of Canada 62: 1-176.

***Betasyrphus:***

Bezzi M (1915) Syrphidae of the Ethiopian region based on material in the collection of the British Museum (Natural History), with descriptions of new genera and species. London, Trustees of the British Museum, pp.29-35. (key)

Curran CH (1938) Records and descriptions of African Syrphidae- II (Diptera). American Museum Novitates 1010: 1-20. (key)

Ssymank A (2010) Review of the species of *Betasyrphus* Matsumura, 1917 (Diptera: Syrphidae) from Madagascar with description of a new species. Zootaxa 2417: 40-50. (key)

***Ceratrichomyia***: (see *Microdon*). Identified by Menno Reemer.

***Chasmomma*:**

Bezzi M (1915) Syrphidae of the Ethiopian region based on material in the collection of the British Museum (Natural History), with descriptions of new genera and species. London, Trustees of the British Museum, pp.102-104.

Curran CH (1939) Records and descriptions of African Syrphidae- III (Diptera). American Museum Novitates 1025: 1-11. (key)

Kassebeer CF (2000) Die Gattung *Chasmomma* Bezzi, 1915 (Diptera, Syrphidae). Dipteron 3: 27-42. (key)

***Eristalinus*:**

Bezzi M (1915) Syrphidae of the Ethiopian region based on material in the collection of the British Museum (Natural History), with descriptions of new genera and species. London, Trustees of the British Museum, pp.77-87. (key)

Curran CH (1927) Article II. – Diptera of the American Museum Congo Expedition. Part I. – Bibionidae, Bombyliidae, Dolichopodidae, Syrphidae and Tryaneidae. Bulletin of the American Museum of Natural History 57: 33-89. (key)

Curran CH (1939) Records and descriptions of Africa Syrphidae – IV (Diptera). American Museum Novitates. 1026: 1-10. (key)

***Eristalis:***

Bezzi M (1915) Syrphidae of the Ethiopian region based on material in the collection of the British Museum (Natural History), with descriptions of new genera and species. London, Trustees of the British Museum, pp.92-94. (key)

Curran CH (1927) Article II. – Diptera of the American Museum Congo Expedition. Part I. – Bibionidae, Bombyliidae, Dolichopodidae, Syrphidae and Tryaneidae. Bulletin of the American Museum of Natural History 57: 33-89. (key)

Curran CH (1939) Records and descriptions of Africa Syrphidae – IV (Diptera). American Museum Novitates. 1026: 1-10. (key)

***Eristalodes:***

Bezzi M (1915) Syrphidae of the Ethiopian region based on material in the collection of the British Museum (Natural History), with descriptions of new genera and species. London, Trustees of the British Museum, pp.87-92. (key)

Curran CH (1927) Article II. – Diptera of the American Museum Congo Expedition. Part I. – Bibionidae, Bombyliidae, Dolichopodidae, Syrphidae and Tryaneidae. Bulletin of the American Museum of Natural History 57: 33-89. (key)

Curran CH (1939) Records and descriptions of Africa Syrphidae – IV (Diptera). American Museum Novitates. 1026: 1-10. (key)

***Eumerus:***

Bezzi M (1915) Syrphidae of the Ethiopian region based on material in the collection of the British Museum (Natural History), with descriptions of new genera and species. London, Trustees of the British Museum, pp.108-116. (key)

Curran CH (1927) Article II. – Diptera of the American Museum Congo Expedition. Part I. – Bibionidae, Bombyliidae, Dolichopodidae, Syrphidae and Tryaneidae. Bulletin of the American Museum of Natural History 57: 33-89. (key)

Curran CH (1938) Records and descriptions of African Syrphidae- I (Diptera). American Museum Novitates 1009: 1-15. (key)

Hervé-Bazin (1913) Syrphidae (Dipt.). Recueillis au Congo Belge par le Dr J. Bequart. I. Genre *Eumerus* Meigen. Revue Zoologique Africaine 3: 68-84. (key)

Hull FM (1964) Diptera (Brachycera): Syrphidae Pp. 442-496. In: Hanström B, Brinck P, Rudebeck G (Eds): South African Animal Life. Results of the Lund University expedition in 1950-1951. 10: 1-496

***Graptomyza:***

Bezzi M (1912) Ditteri raccolti da Leonardo Fea durante il suo viaggio dell’Africa occidentale. Parte Ia: Syrphidae. Annali del Museo Civico di Storia Naturale di Genova 5: 400-443. (key)

Bezzi M (1915) Syrphidae of the Ethiopian region based on material in the collection of the British Museum (Natural History), with descriptions of new genera and species. London, Trustees of the British Museum, pp.55-61. (key)

Curran CH (1927) Article II. – Diptera of the American Museum Congo Expedition. Part I. – Bibionidae, Bombyliidae, Dolichopodidae, Syrphidae and Tryaneidae. Bulletin of the American Museum of Natural History 57: 33-89. (key)

Curran CH (1938) Records and descriptions of African Syrphidae- I (Diptera). American Museum Novitates 1009: 1-15. (key)

Whittington AE (1992) Revision of the Afrotropical species of *Graptomyza* Wiedemann (Diptera: Syrphidae: Volucellini). Annals of the Natal Museum 33: 209-269. (key)

Whittington AE (1994) Distribution and conservation of Afrotropical *Graptomyza* Wiedemann, with a new species description (Diptera: Syrphidae: Volucellini). Biodiversity Conservation 3: 716-733. (key)

***Melanostoma:***

Bezzi M (1915) Syrphidae of the Ethiopian region based on material in the collection of the British Museum (Natural History), with descriptions of new genera and species. London, Trustees of the British Museum, pp.18-21. (key)

Curran CH (1938) Records and descriptions of African Syrphidae- II (Diptera). American Museum Novitates 1010: 1-20. (key)

Dirickx HG (2001) Notes sur le genre *Melanostoma* Schiner, 1860 (Diptera, Syrphidae) à Madagascar et les îles voisines avec description de cinq espèces nouvelles. Revue Suisse de Zoologie 108 : 993-1029. (key)

***Mesembrius:***

Bezzi M (1915) Syrphidae of the Ethiopian region based on material in the collection of the British Museum (Natural History), with descriptions of new genera and species. London, Trustees of the British Museum, pp.94-98. (key)

Curran CH (1927) Article II. – Diptera of the American Museum Congo Expedition. Part I. – Bibionidae, Bombyliidae, Dolichopodidae, Syrphidae and Tryaneidae. Bulletin of the American Museum of Natural History 57: 33-89. (key)

Curran CH (1939) Records and descriptions of African Syrphidae- III (Diptera). American Museum Novitates 1025: 1-11. (key)

***Metadon:*** (see *Microdon*). Identified by Menno Reemer.

***Microdon:*** see also Note 2. Identified by Menno Reemer.

Bezzi M (1915) Syrphidae of the Ethiopian region based on material in the collection of the British Museum (Natural History), with descriptions of new genera and species. London, Trustees of the British Museum, pp.119-136. (key)

Hervé-Bazin (1913) Syrphidae (Dipt.). Recueillis au Congo Belge par le Dr J. Bequart. III. Genre *Microdon* Meigen. Revue Zoologique Africaine 3 :68-84. (key)

Curran CH (1938) Records and descriptions of African Syrphidae- I (Diptera). American Museum Novitates 1009: 1-15. (key)

Reemer M, Ståhls G (2013) Phylogenetic relationships of Microdontinae (Diptera: Syrphidae ) based on molecular and morphological characters. Systematic Entomology 38: 661-688.

Reemer M, Ståhls G (2013) Generic revision and species classification of the Microdontinae (Diptera, Syrphidae). ZooKeys 288: 1-213. (key)

***Paragus:***

Bezzi M (1915) Syrphidae of the Ethiopian region based on material in the collection of the British Museum (Natural History), with descriptions of new genera and species. London, Trustees of the British Museum, pp.11-15. (key)

Curran CH (1938) Records and descriptions of African Syrphidae- II (Diptera). American Museum Novitates 1010: 1-20. (key)

Kassebeer CF (1998) Eine obskure *Pandasyopthalmus* Stuckenberg, 1954 mit Flügelzeichnung aus Westafrika (Diptera, Syrphidae). Dipteron 1: 1-9.

Kassebeer CF (1999) Die Gattung *Paragus* Latreille, 1804 (Diptera, Syrphidae) in der Elfenbeinküste. Dipteron 2: 31-44.

Kassebeer CF (1999) Die Gattung *Paragus* Latreille, 1804 (Diptera, Syrphidae) auf Madagaskar, den Komoren und den Maskarenen. Dipteron 2: 75-92.

Stuckenberg BR (1954) Studies on *Paragus* with descriptions of new species (Diptera Syrphidae). Revue de Zoologie et de Botanique Africaines 49: 97-139. (key)

Stuckenberg BR (1954) The *Paragus serratus* complex, with descriptions of new species (Diptera: Syrphidae). The Transactions of the Royal Entomological Society of London 105: 393-422. (key)

***Paramixogaster:*** (see *Microdon*). Identified by Menno Reemer.

***Phytomia:***

Bezzi M (1915) Syrphidae of the Ethiopian region based on material in the collection of the British Museum (Natural History), with descriptions of new genera and species. London, Trustees of the British Museum, pp.65-76. (key)

Curran CH (1927) Article II. – Diptera of the American Museum Congo Expedition. Part I. – Bibionidae, Bombyliidae, Dolichopodidae, Syrphidae and Tryaneidae. Bulletin of the American Museum of Natural History 57: 33-89. (key)

Curran CH (1939) Records and descriptions of Africa Syrphidae – IV (Diptera). American Museum Novitates. 1026: 1-10. (key)

***Polybiomyia:***

Hervé-Bazin (1913) Syrphidae (Dipt.). Recueillis au Congo Belge par le Dr J. Bequart. II. Genre *Cerioides* Rond.. Revue Zoologique Africaine 3: 85-95. (key)

Curran CH (1938) Records and descriptions of African Syrphidae- I (Diptera). American Museum Novitates 1009: 1-15. (key)

Thompson CF (2013) A new Afrotropical ceriodine flower fly with an overview of the group (Diptera: Syrphidae, Cerioidini). Entomologist’s Monthly Magazine 149: 71-77. (key)

***Rhingia:***

Bezzi M (1915) Syrphidae of the Ethiopian region based on material in the collection of the British Museum (Natural History), with descriptions of new genera and species. London, Trustees of the British Museum, pp.50-55. (key)

Curran CH (1927) Article II. – Diptera of the American Museum Congo Expedition. Part I. – Bibionidae, Bombyliidae, Dolichopodidae, Syrphidae and Tryaneidae. Bulletin of the American Museum of Natural History 57: 33-89. (key)

Curran CH (1939) Records and descriptions of Africa Syrphidae – IV (Diptera). American Museum Novitates. 1026: 1-10. (key)

Speiser P (1910) 5. Cyclorhapha, Aschiza. Syrphidae. In: Sjöstedt Y (Ed). Wissenschaftliche Ergebnisse der Schwedischen Zoologischen Expedition nach dem Kilimandjaro, dem Meru und den Umgebenden Massaisteppen Deutsch-Ostafrikas. 2: 15-131.

***Senaspis:***

Bezzi M (1912) Ditteri raccolti da Leonardo Fea durante il suo viaggio dell’Africa occidentale. Parte Ia: Syrphidae. Annali del Museo Civico di Storia Naturale di Genova 5: 400-443. (key)

Bezzi M (1915) Syrphidae of the Ethiopian region based on material in the collection of the British Museum (Natural History), with descriptions of new genera and species. London, Trustees of the British Museum, pp.61-65. (key)

Curran CH (1927) Article II. – Diptera of the American Museum Congo Expedition. Part I. – Bibionidae, Bombyliidae, Dolichopodidae, Syrphidae and Tryaneidae. Bulletin of the American Museum of Natural History 57: 33-89. (key)

Curran CH (1939) Records and descriptions of Africa Syrphidae – IV (Diptera). American Museum Novitates. 1026: 1-10. (key)

***Syritta:***

Bezzi M (1915) Syrphidae of the Ethiopian region based on material in the collection of the British Museum (Natural History), with descriptions of new genera and species. London, Trustees of the British Museum, pp.104-108. (key)

Curran CH (1939) Records and descriptions of African Syrphidae- III (Diptera). American Museum Novitates 1025: 1-11. (key)

Lyneborg L, Barkemeyer W (2005) The Genus *Syritta*: A World Revision of the Genus *Syritta* Le Peletier & Serville, 1828 (Diptera: Syrphidae). Entomonograph Volume 15, Apollo Books, Denmark.

***Toxomerus:***

Borges ZM, Couri MS (2009) Revision of *Toxomerus* Macquart, 1855 (Diptera: Syrphidae) from Brazil with synonymic notes, identification key to the species and description of three new species. Zootaxa 2179: 1–72. (key)

Curran CH (1930) New Diptera belonging to the genus *Mesogramma* Loew (Syrphidae). American Museum Novitates 405: 1–14. (key)

Hull FM (1943) The genus *Mesogramma.* Entomológica Americana 23: 1–41. (key)

Metz MA, Thompson FC (2001) A revision of the larger species of *Toxomerus* (Diptera: Syrphidae) with description of a new species. Studia dipterologica 8: 225–256. (key)

Thompson FC (1981) The flower flies of the West Indies (Diptera: Syrphidae). Memoirs of the Entomological Society of Washington 9: 1–200. (key)

Thompson FC (1999) A key to the genera of the flower flies (Diptera: Syrphidae) of the Neotropical Region including descriptions of new genera and species and a glossary of taxonomic terms. Contributions on Entomology 3: 321–378. (key)
